# Earth‐Abundant Tin Sulfide‐Based Photocathodes for Solar Hydrogen Production

**DOI:** 10.1002/advs.201700362

**Published:** 2017-10-16

**Authors:** Wei Cheng, Nirala Singh, Will Elliott, Joun Lee, Alan Rassoolkhani, Xuejun Jin, Eric W. McFarland, Syed Mubeen

**Affiliations:** ^1^ Department of Chemical and Biochemical Engineering University of Iowa Iowa City IA 52242 USA; ^2^ Department of Chemical Engineering University of California Santa Barbara CA 93106 USA; ^3^ Department of Chemistry University of California Santa Barbara CA 93106 USA; ^4^ School of Materials Science and Engineering Shanghai Jiao Tong University Shanghai 200240 China

**Keywords:** artificial photosynthesis, electrocatalyst, hydrogen production, photocathode, tin sulfide

## Abstract

Tin‐based chalcogenide semiconductors, though attractive materials for photovoltaics, have to date exhibited poor performance and stability for photoelectrochemical applications. Here, a novel strategy is reported to improve performance and stability of tin monosulfide (SnS) nanoplatelet thin films for H_2_ production in acidic media without any use of sacrificial reagent. P‐type SnS nanoplatelet films are coated with the *n*‐CdS buffer layer and the TiO_2_ passivation layer to form type II heterojunction photocathodes. These photocathodes with subsequent deposition of Pt nanoparticles generate a photovoltage of 300 mV and a photocurrent density of 2.4 mA cm^−2^ at 0 V versus reversible hydrogen electrode (RHE) for water splitting under simulated visible‐light illumination (λ > 500 nm, *P*_in_ = 80 mW cm^−2^). The incident photon‐to‐current efficiency at 0 V versus RHE for H_2_ production reach a maximum of 12.7% at 575 nm with internal quantum efficiency of 13.8%. The faradaic efficiency for hydrogen evolution remains close to unity after 6000 s of illumination, confirming the robustness of the heterojunction for solar H_2_ production.

Artificial photosynthesis provides means for directly converting photon energy in sunlight to chemical potential energy by producing molecular products, which can be later used as fuels and chemicals. Traditionally, these systems are based on solid‐state semiconductor‐based photoelectrodes, which when appropriately engineered, separate and deliver photogenerated carriers to electrocatalysts for carrying out the desired chemistry.^1^, ^2^, ^3^, ^4^, ^5^, ^6^, ^7^, ^8^, ^9^, ^10^, ^11^, ^12^, ^13^, ^14^ Recent efforts have focused on developing cost‐effective and stable light‐absorber materials with relatively narrow bandgap (that absorbs light over a broad spectral range (300–900 nm)) for maximizing stored solar energy, an absolute necessity for large‐scale deployment.

Metal chalcogenide semiconducting materials from IV to VI groups have emerged as one such group of promising light‐absorber materials for photoelectrochemical (PEC) applications because of their narrow bandgap, earth abundance, and low materials processing cost.^15^, ^16^ Specifically, tin monosulfide (SnS) has been studied for water splitting because of its narrow optical bandgap of 1.1–1.4 eV and favorable conduction band energetics for H_2_ evolution.^17^, ^18^, ^19^, ^20^, ^21^, ^22^, ^23^ However, poor film quality and instability in the PEC environment of the cell have so far limited their application to systems with sacrificial reagents.^24^, ^25^

In this study, we report PEC properties of crystalline nanoplatelet SnS photocathodes fabricated using low‐cost, solution‐processable technique for solar H_2_ production. First, using a regenerative PEC system (no net chemical change), we demonstrate that the SnS nanoplatelet thin films can serve as efficient photocathodes with a maximum photocurrent density of 12 mA cm^−2^. We then report a facile strategy to fabricate type II heterojunction H_2_‐producing photocathodes, consisting of the *p*‐SnS/*n*‐CdS/*n*‐TiO_2_/Pt, and demonstrate for the first time, active (2.4 mA cm^−2^ at 0 V vs reversible hydrogen electrode (RHE)) H_2_ production in acidic media with the highest reported IPCE for these materials to date (Please see Table S1 in the Supporting Information which compares present work with other published reported values).^26^

The SnS thin films were synthesized on fluorine‐doped tin oxide (FTO)‐coated glass using a modified chemical bath deposition (CBD) method.^20^, ^27^ Detailed accounts of the synthetic method are given in the Experimental Section. **Figure** **1**a,b shows top and cross‐sectional scanning electron microscopy (SEM) images of SnS films deposited on the FTO substrate. The films produced using the above approach showed nanoplatelet morphology with individual platelets of thickness 12.3 ± 1.0 nm (Figure 1a, inset) and a total height of ≈600 nm. The nanoplatelet morphology is an effective structure for light absorption and carrier extraction, by collecting carriers orthogonal to the direction of the incident light. Areas over 5.0 cm^2^ were frequently produced with no delamination of films observed in any of the samples. Transmission electron microscopy (TEM) (Figure 1c) and X‐ray diffraction (XRD) measurements (Figure 1d) revealed highly crystalline orthorhombic‐structured tin monosulfide films with strong 〈111〉 orientation.^28^ Bandgap and direct/indirect transitions of the SnS films were determined using the Tauc equation, given by (*αhν*)*^n^* = *k*(*hν* − *E_g_*).^29^ Where, α is the measured optical absorption coefficient, *hν* is the photon energy, *E*_g_ is the bandgap, and *k* is the proportionality constant. A linearity of the plot (*αhν*)*^n^* versus *hν* was obtained for *n* = ½, an indicative of indirect transition with *x*‐axis intercept of 1.1 eV, indicating the bandgap of the synthesized films (Figure 1e).

**Figure 1:**
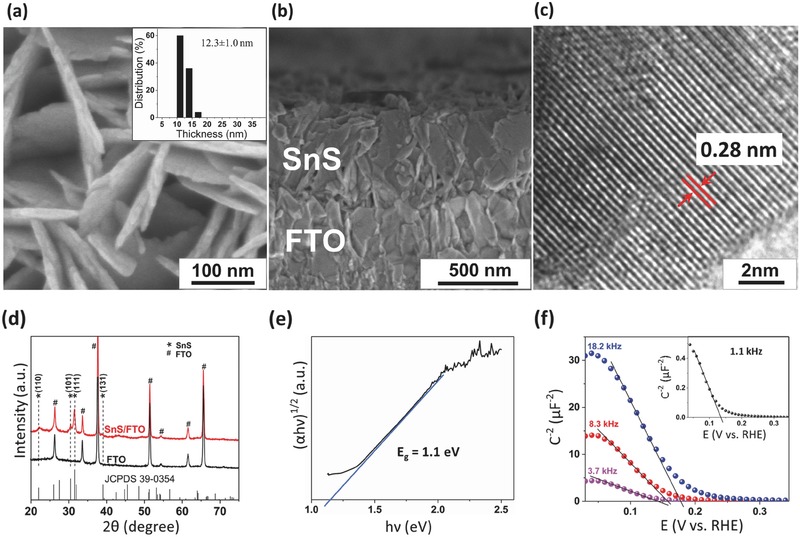
Top‐view a) and cross‐sectional b) SEM images of the SnS film deposited on FTO glass substrate. The inset of (a) shows thickness distribution of SnS nanoplatelets. c) Lattice‐resolved high‐resolution TEM image of an SnS nanoplatelet showing *d*‐spacing of 0.28 nm corresponding to SnS (111). d) XRD spectrum of FTO glass, the SnS film deposited on FTO substrate with JCPDS card (No. 39‐0354) of Herzenbergite SnS. # and * indicate peaks of FTO and SnS, respectively. e) Tauc analysis of optical absorption spectra showing an indirect bandgap of 1.1 eV for SnS and f) Mott–Schottky plots measured for the SnS film in 0.1 m Na_2_S + 0.1 m S (pH 9) for different frequencies (1.1 kHz (black trace, inset), 3.7 kHz (pink trace), 8.3 kHz (red trace), and 18.2 kHz (blue trace)).

To determine the conductivity type and carrier concentration of SnS films, Mott–Schottky plots (1/*C* ^2^ vs *E*) were constructed in 0.1 m Na_2_S + 0.1 m S (pH 9) (Figure 1f). *C* is the differential capacitance, and *E* is the applied potential with respect to RHE. The SnS films showed a region of ≈300 mV where 1/*C* ^2^ linearly increased with decreasing potential under reverse bias conditions, indicating p‐type behavior. The Mott–Schottky plots exhibited frequency dispersion indicating surface states, a trend observed in most nanostructured films.^30^, ^31^, ^32^ The donor concentration of the films (measured from the slope of the 1/*C* ^2^ vs *E* plots) ranged from 3.6 × 10^17^ cm^−3^ (at 18.2 kHz) to 1.57 × 10^19^ cm^−3^ (at 1.1 kHz), based on a relative dielectric constant of 19.5.^33^ The flat‐band potential (*E*_fb_) obtained from the x‐intercept of the 1/*C* ^2^ versus *E* plots, showed narrow ranges from 0.17 V versus RHE (at 1.1 kHz) to 0.20 V versus RHE at (18.2 kHz). The p‐type behavior and *E*_fb_ obtained from Mott–Schottky measurements matched with previously reported theoretical estimations for SnS.^23^

The PEC properties of the SnS photocathode were first tested in the presence of regenerative polysulfide redox couple (S_2_^2−^/HS^−^), shown in **Figure** **2**. In a PEC system with the regenerative redox couple, the semiconductor electrode and counter electrode perform the same electrochemical reaction, but in the opposite direction. That is, the photon energy is converted into electrical energy with no net chemical change to the system. The redox couple most often serves to stabilize the semiconductor from the PEC corrosion and provides a rapid and convenient way to evaluate the structure–activity relationship of the semiconductor electrode without the complexity of surface passivation. The photocurrents were measured using a three‐electrode PEC cell with Pt wire as the counter electrode and a saturated calomel electrode (SCE) as the reference electrode. A light fluence of 100 mW cm^−2^ obtained from 300 W xenon lamp fitted with appropriate AM 1.5 and IR filters were used for all regenerative PEC runs, (see the Experimental Section for details). A chopped illumination was used for the measurements, so the dark and photocurrents could be monitored simultaneously.

**Figure 2:**
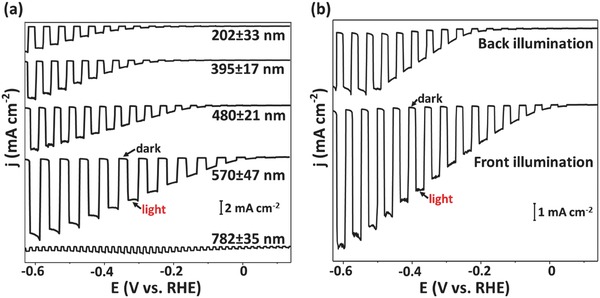
PEC characterization of SnS films in 0.1 m Na_2_S + 0.1 m S (pH 9) under chopped simulated sunlight (AM1.5). a) Current density–potential (*j*–*E*) characteristics of the SnS films synthesized with different thicknesses (202 ± 33, 395 ± 17, 480 ± 21, 573 ± 47, 782 ± 35 nm) illuminated from the front side. b) *j*–*E* plots of SnS films with a thickness of ≈600 nm under back illumination and front illumination.

Figure 2a shows typical current–potential curves obtained in S_2_^2−^/HS^−^ electrolyte. Negligible cathodic currents were observed for the *p*‐SnS in the dark for all potential ranges, as expected for a p‐type semiconductor with a low density of electrons (minority carriers) at the surface. Under illumination, the cathodic currents increased substantially since electrons from the valence band were promoted by the absorbed light into the conduction band and were available for reduction reactions. The measured photocurrents increased with increasing thickness of the deposited film (Figure 2a), which was controlled by changing the CBD time (Figure S1, Supporting Information). SnS films of a thickness of ≈600 nm were sufficient to absorb all incident light with wavelengths <1100 nm (Figure S2, Supporting Information; assuming no reflection loss), yielding a photocurrent density of 12 mA cm^−2^ under 100 mW cm^−2^ front illumination (AM 1.5 solar spectrum). This is the highest reported photocurrent to date for SnS‐based photocathodes in the presence of aqueous redox electrolytes.^34^ Thicknesses beyond 600 nm (≈750 nm) yielded low photocurrent density. This could be due to a majority carrier transport limitation due to increased distance of the majority carriers to transport through the film. The photocurrent density was lower when the sample was illuminated from the back than from the front (Figure 2b). The decrease in the cathodic photocurrent under back illumination can be attributed to minority carrier recombination as the minority carriers that are generated near the back contact are further from the semiconductor–electrolyte interface.

The optimized SnS photocathodes were then tested for the PEC H_2_ production in a nitrogen‐purged 0.5 m H_2_SO_4_, which was the electrolyte used for all H_2_ production experiments. For all experiments, a Pt wire served as the counter electrode and an SCE served as the reference electrode (see the Experimental Section for details). Nitrogen was purged to remove oxygen and to eliminate the possibility of the oxygen reduction reaction. **Figure** **3**a shows the chopped photocurrent density transient (*j*–*t* plot) at 0 V versus RHE (*E*_RHE_ =*E*_SCE_ + 0.241 V + 0.059*pH) for bare *p*‐SnS coated with 2 nm electron beam‐deposited Pt nanoparticles. The Pt nanoparticles served as the catalyst for H_2_ evolution. Under AM 1.5 illumination, bare SnS films with Pt catalyst produced an initial cathodic photocurrent density of 2.9 mA cm^−2^. However, the photocurrents decreased substantially with time, indicating chemical and PEC degradation of SnS films in the acidic environment of the electrochemical cell.^35^ Discoloration of the films could be seen visibly with complete disappearance of the deposit after 30 min.

**Figure 3:**
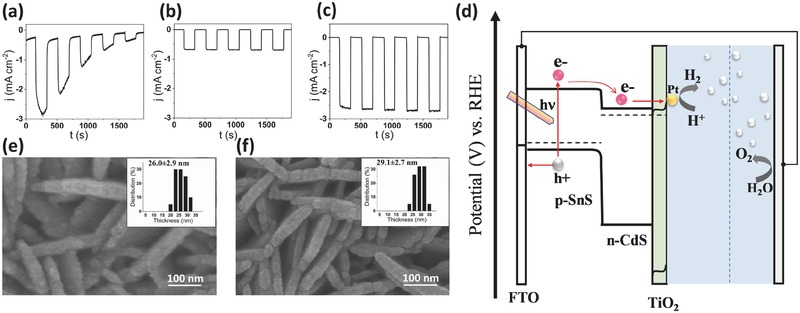
Current density–time (*j*–*t*) characteristics of a) SnS/Pt, b) SnS/TiO_2_/Pt, and c) *p*‐SnS/*n*‐CdS/*n*‐TiO_2_/Pt in 0.5 m H_2_SO_4_ under chopped simulated sunlight. d) The energy band diagram of the *p*‐SnS/*n*‐CdS/*n*‐TiO_2_/Pt for PEC water splitting. Top‐view SEM images of e) SnS/CdS and f) SnS/CdS/TiO_2_ deposited on FTO substrate with corresponding thickness histograms shown in the inset.

To protect the SnS films from degradation, 2 nm mass equivalent thick TiO_2_ films were deposited on the top of SnS using atomic layer deposition (ALD) (see the Experimental Section and Figure S3 in the Supporting Information for more details). Thin conformal layers of TiO_2_ have been shown to effectively protect underlying semiconductors such as Cu_2_O and Si from photocorrosion while serving as an effective electron filter due to its large valence band offset.^36^, ^37^ The number of cycles to deposit 2 nm TiO_2_ film was obtained from precalibrated ellipsometry data (Figure S3, Supporting Information). Chopped photocurrent density transient from the resulting SnS/TiO_2_ films with 2 nm electron‐beam‐deposited Pt nanoparticles is shown in Figure 3b. Stable photocurrent densities were obtained after the addition of the TiO_2_ layer with a maximum value 0.6 mA cm^−2^ for the champion device. As explained below, a buffer layer of CdS (Figure S4, Supporting Information) between SnS and TiO_2_ layers was required to increase the photocurrent density to 2.4 mA cm^−2^ (Figure 3c).

The enhanced photocurrents with the introduction of *n*‐CdS can be understood qualitatively with the aid of the schematic band diagram shown in Figure 3d. Previous reported experimental and theoretical calculations indicate that a staggered type II heterojunction is formed between the *p*‐SnS and *n*‐CdS.^38^, ^39^ Staggered type II heterojunction implies an unrestricted flow of electrons from p‐type light‐absorber unit to the n‐type buffer layer due to appropriate interfacial energetics. To determine the nature of the heterojunction and its effect on flat‐band potential and carrier density, Mott–Schottky plots were constructed (Figure S9, Supporting Information) for SnS/CdS and SnS/CdS/TiO_2_ films in 0.1 m Na_2_S + 0.1 m S (pH 9). The Mott–Schottky results show that the flat‐band potential shifts are positive with the addition of CdS and TiO_2_ layers. Shifts in the flat‐band potential could be due to changes either in the donor density or in the helmholtz potential drop across the semiconductor/electrolyte interface. Since the charge carrier density of the heterojunction films was comparable to bare SnS films (Figure S9, Supporting Information), the positive shift to the flat‐band potential indicates that the potential drop across electrolyte interface is no longer determined by the *p*‐SnS but by the heterojunction formed between SnS/CdS/TiO_2_ layers. Figure 3e,f shows top‐view SEM images of SnS/CdS on and SnS/CdS/TiO_2_ films with the inset showing a histogram of platelet thickness. The addition of the CdS layer increased the individual platelet thickness from 12.3 ± 1.0 nm (Figure 1a) to 26.0 ± 2.9 nm (Figure 3e, inset), and to 29.1 ± 2.7 nm (Figure 3f, inset) with subsequent addition of the TiO_2_ layer. X‐ray photoelectron spectroscopy (XPS; Figures S5 and S6, Supporting Information) and depth profiling using secondary ion mass spectrometry (Figures S7 and S8, Supporting Information) were used to confirm the chemical composition and heterojunction nature of the films.

**Figure** **4**a shows typical photocurrent density (*j*)–potential (*E*) curves for hydrogen production from *p*‐SnS/*n*‐CdS/*n*‐TiO_2_/Pt films and *p*‐SnS/Pt films on FTO substrate illuminated with simulated visible solar spectrum (>500 nm and light intensity 80 mW cm^−2^). Wavelengths greater than 500 nm were chosen for these measurements to eliminate any photocurrents originating from underlying CdS (*E*_g_ ≈ 2.5 eV) and TiO_2_ (*E*_g_ ≈ 3.2 eV) layers. Upon varying the applied voltage, the *p*‐SnS/*n*‐CdS/*n*‐TiO_2_/Pt photocathodes showed a significant enhancement in the PEC activity with the onset of photocurrents beginning at 0.3 V versus RHE (a maximum open‐circuit voltage of ≈0.45 V is expected based on the Fermi level difference between *p*–*n* SnS/CdS^38^, ^39^). At 0 V versus RHE, a photocurrent density of 2.4 mA cm^−2^ was obtained with no evidence of material degradation over the experimental duration. This is the highest photocurrent achieved to date with the *p*‐SnS as light‐absorber material for H_2_ production (see Table S1 in the Supporting Information). We stress that no sacrificial reagent was used for H_2_ production.

**Figure 4:**
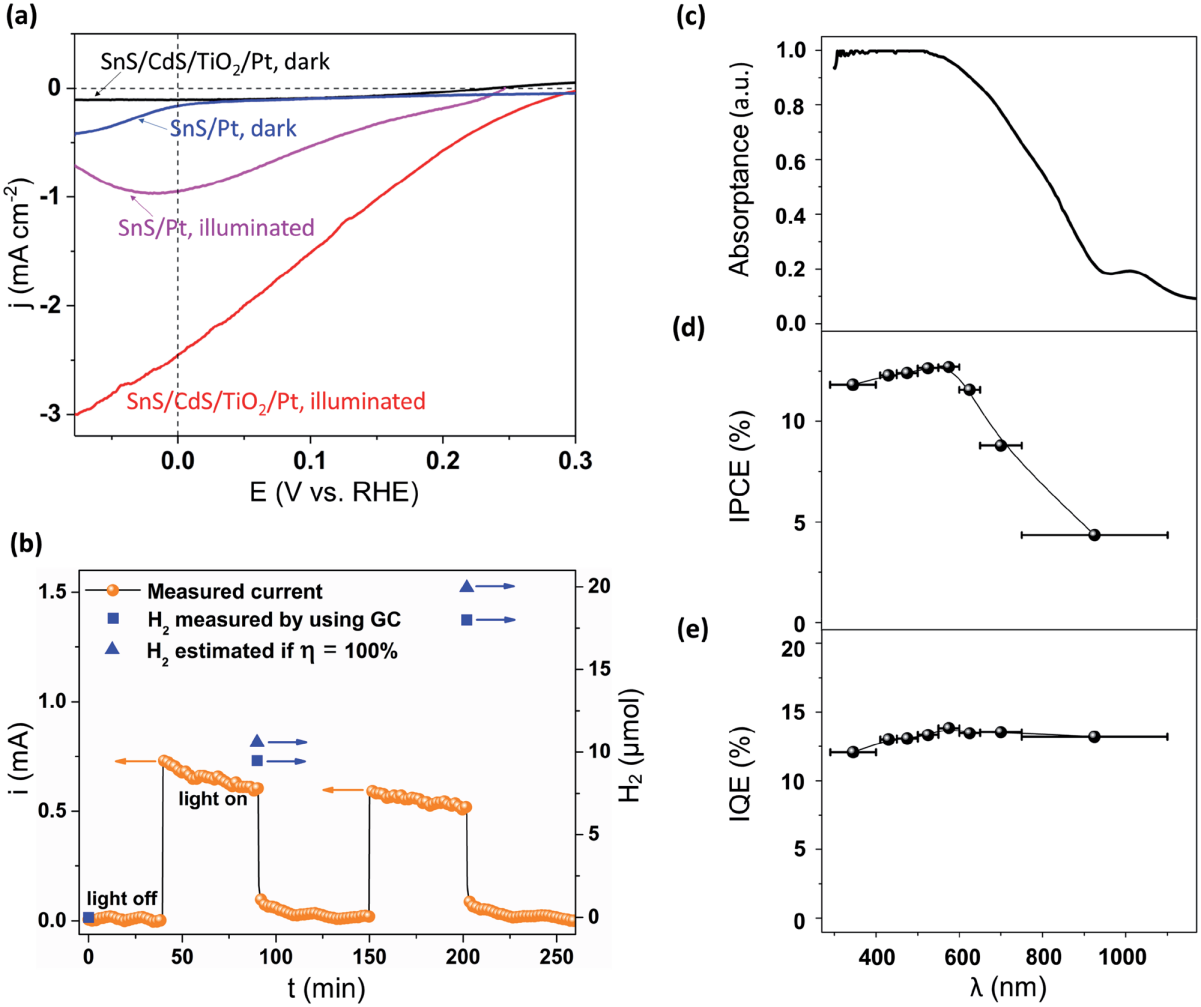
a) *j*–*E* characteristics of *p*‐SnS/*n*‐CdS/*n*‐TiO_2_/Pt and *p*‐SnS/Pt electrodes in 0.5 m H_2_SO_4_ solution in dark and under simulated visible sunlight (>500 nm, 80 mW cm^−2^, red line). b) Faradaic efficiency measurement for hydrogen production through current–time characteristic (orange sphere) of the heterojunction photocathode in 0.5 m H_2_SO_4_, illuminated by simulated visible sun light (>500 nm, 80 mW cm^−2^). The electrode area was 0.23 cm^2^. The amount of H_2_ measured by GC (blue square) and the estimated amount of H_2_ if Faradaic efficiency was 100% (blue triangle) is also shown in panel (b). c) The absorption spectrum of the *p*‐SnS/*n*‐CdS/*n*‐TiO_2_/Pt film. d) The incident photon‐to‐current efficiency (IPCE) and e) IQE of the *p*‐SnS/*n*‐CdS/*n*‐TiO_2_/Pt photocathode measured under simulated sunlight at 0 V versus RHE in 0.5 m H_2_SO_4_.

To evaluate electrode stability and the reaction products, gas chromatography (GC) was employed to quantify the amount of H_2_ produced. We continued the PEC experiment for over 6000 s at 0 V versus RHE under visible‐light illumination (λ > 500 nm) for *p*‐SnS/*n*‐CdS/*n*‐TiO_2_/Pt photocathodes. Before the experiment, the solution was purged with nitrogen to eliminate currents due to oxygen reduction reaction. The evolved H_2_ product as measured by GC (blue squares) and the amount of estimated H_2_ for 100% faradaic efficiency (blue triangles) are shown in Figure 4b. We were able to obtain 90% Faradaic efficiency after 2 h. We believe the 10% loss in faradaic efficiency is due to experimental error and not due to photocorrosion of the samples. We base the above argument based on the following calculation: For 600 nm thick films, assuming two electrons for reducing SnS (photocorrosion), the amount of charge required to consume SnS completely is 0.4 Coulombs cm^−2^, while the total amount of charge not accounted due to 10% efficiency loss is 1.73 Coulombs cm^−2^. The above calculation corroborates that the measured photocurrent (represented in orange spheres) is indeed due to the PEC water reduction producing H_2_.

To further understand the carrier transport process, incident photon‐to‐current efficiency (IPCE) and internal quantum efficiency (IQE) measurements were carried out on *p*‐SnS/*n*‐CdS/*n*‐TiO_2_/Pt films (Figure 4c–e). The absorptance of the heterojunction photocathode (Figure 4c) was measured using an integrating sphere, which shows the band‐edge absorption onset at ≈1100 nm. The IPCE spectrum of the *p*‐SnS/*n*‐CdS/*n*‐TiO_2_/Pt film in Figure 4d follows the distinct excitonic features of the absorption spectrum of the *p*‐SnS covered by the *n*‐CdS and *n*‐TiO_2_, with the IPCE reaching a plateau at 12.7% near 575 nm. The identical trend between the PEC action spectrum and the absorption spectrum of the device is strong evidence that SnS dominates the initiating photoprocesses. Finally, the photocurrents produced are determined largely by the photon capture efficiency, and by the IQE, defined as the number of photoexcited carriers in the semiconductor contributing to photocurrent per absorbed photon (Figure 4e). The IQE was determined by scaling IPCE by the fraction of incident photons absorbed by the photocathode unit at a given wavelength. For *p*‐SnS/*n*‐CdS/*n*‐TiO_2_/Pt devices, the IQE remained constant at ≈13.0% across the entire visible wavelength range.

In summary, we report a *p*‐SnS‐based heterojunction photocathode that can function as an efficient hydrogen‐evolving electrode for proton reduction. Cathodic photocurrents of up to 12 mA cm^−2^ were obtained using S_2_^2−^/HS^−^ redox couple. Using a serially staggered type II‐band offset architecture, a record H_2_‐evolving photocurrents up to 2.4 mA cm^−2^ at 0 V versus RHE was obtained with no sacrificial reagent. The H_2_ production currents were stable for 6000 s under illumination, and the faradaic efficiency was effectively 100% indicating the robustness of the heterojunction for H_2_ production in an acidic environment.

## Experimental Section

*CBD of SnS*: FTO glass slides were used as a substrate for the deposition of SnS films. All chemicals were purchased from Sigma‐Aldrich. Before the deposition of the films, the FTO substrates were ultrasonically cleaned in acetone, isopropanol, and DI water for 10 min each. To prepare the growth solution for CBD, first, 0.1 m SnCl_2_·2H_2_O (purity ≥ 99.995%) was completely dissolved in 20 mL of acetone. This was followed by an addition of 0.75 m of triethanolamine (TEA, purity ≥99.0%), 20 mL of commercial ammonium hydroxide (28–31%), and 20 mL of DI water. Sufficient time for mixing (≈4 min) was allowed between the additions of TEA, ammonium hydroxide, and water. The solution was stirred for another 4–5 min before addition of 0.1 m of thioacetamide (purity ≥ 99.0%). DI water was added to make the volume of total growth solution equal to 200 mL. The growth solution was then kept at 85 °C in an oil bath. FTO substrates were then immersed in the solution with their conductive sides facing downward angling 60° to the bottom of the beaker. After the CBD deposition for required amount of time (0.5–2 h), the substrates were taken out of the growth solution, rinsed with DI water, and then left to dry naturally in the air. All samples were annealed in nitrogen for 1 h at 200 °C prior to PEC runs.

*CBD of CdS*: CdS was deposited on SnS films following well‐established CBD recipes previously reported.^40^ The solution for CBD growth of CdS included 0.01 m of cadmium sulfate, 0.0125 m of ammonium sulfate, 0.075 m of thiourea, and 0.025 m of potassium iodide in 100 mL DI water. All chemicals were purchased from Sigma‐Aldrich. The pH of the chemical bath was adjusted to 11.5 by adding ammonium hydroxide. The CdS growth solution, under mild magnetic stirring, was heated to 80 °C in an oil bath. The CBD grown SnS films were vertically submerged into the CdS growth solution. After 20 min of CBD, the samples were taken out of the solution, rinsed with DI water, and dried naturally in the air. Figure S3 in the Supporting Information shows the cross‐sectional SEM image of the SnS/CdS layer.

*ALD of TiO_2_*: The ALD of TiO_2_ was carried out in an Oxford FlexAL tool at 200 °C using tetrakis(dimethylamino)titanium and water as precursor and reactant, respectively.^36^ After ALD deposition, the samples were annealed at 200 °C under a nitrogen atmosphere before e‐beam evaporation of platinum. A mass equivalent thickness of 2 nm of Pt was deposited as the hydrogen‐evolution catalyst for all the samples.

*Structural Characterization*: The morphology of the films was characterized using a field emission scanning electron microscope (Hitachi S‐4800) and a field emission transmission electron microscope (JEOL JEM 2100F, FEG). XRD patterns were acquired with a Philips X'PERT MPD diffractometer, using Cu Ka radiation (1.5405 Å) over a range of 2θ = 20°–60° at a scan rate of 4° min^−1^. XPS analysis was carried out using Kratos Axis Ultra X‐ray photoelectron spectrometer with concentric hemispherical electron energy analyzers combined with the established delay‐line detector. The incident radiation monochromatic Al Kα X‐ray (1486.6 eV) at 150 W (accelerating voltage 15 kV, emission current 10 mA) was projected 45° to the sample surface and the photoelectron data were collected at takeoff angle of θ = 90°. The absolute energy scale was calibrated to Cu 2p_3/2_ peak binding energy at 932.6 eV using sputter etched 99.9999% pure copper foil. The base pressure in the analysis chamber was maintained at 1.0 × 10^−9^ torr. Low‐energy electrons were used for charge compensation to neutralize the sample. Survey scans were taken at pass energy of 160 eV and carried out over 1200 to −5 eV binding energy range with 1.0 eV steps and a dwell time of 200 ms. High resolution scans of Sn 3d, Cd 3d, and S 2p were taken at pass energy of 20 eV with 0.1 eV steps and a dwell time of 1000 ms. The spectra analyses were carried out using CasaXPS version 2.3.18PR1.0. Shirley type background was routinely used to account for inelastically scattered electrons that contributed to the broad background. Transmission‐corrected RFS/Kratos library relative sensitivity factors were used for elemental quantification. The spectra were calibrated using adventitious carbon C 1s peak at 284.8 eV. Dynamic secondary ion mass spectroscopy was performed with a Cameca IMS 7f‐Auto system using an oxygen (O_2_^+^) primary ion beam at an impact energy of 2 kV. Typical background pressure in the system was 1 × 10^−9^ mbar. The crater size was 175 µm, and secondary ions were monitored from a 63 µm diameter circle in the center to avoid crater‐edge effects. Various secondary ion species were monitored, and data were recorded at intervals of ≈1 nm depth for each element. Depth scales were quantified using a contact profilometer.

*PEC Measurements Using Polysulfide Redox Couple*: PEC measurements for the redox couple of HS^−^/S_2_^2−^ were carried out in a three‐electrode configuration with bare SnS thin films as the working electrode. A 300 W xenon lamp source fitted with AM 1.5 and IR filter was used to simulate sunlight with an intensity of 100 mW cm^−2^ measured using a thermopile sensor (Newport). All linear sweep voltammetry measurements were carried out at a scan rate of 20 mV s^−1^. For the redox couple of HS^−^/S_2_^2−^, the electrolyte composition was 0.1 m Na_2_S + 0.1 m S (pH = 9), with SCE and Pt wire as reference and counter electrode, respectively.

*PEC Measurements for Hydrogen Production*: For PEC studies of H_2_ production, 0.5 m H_2_SO_4_ was used as an electrolyte with SCE and Pt wire as a reference and counter electrodes, respectively. The scan rate for the linear sweep voltammetry and the cyclic voltammetry was 10 mV s^−1^. The photoelectrodes were illuminated with simulated AM 1.5 solar spectrum (Newport Xe Arc Lamp Source) equipped with liquid IR filter and a cut‐off filter (λ > 500 nm). The incident light intensity was measured to be 80 mW cm^−2^. Photocurrent stability tests were carried out by measuring photocurrents under chopped light illumination at a fixed electrode potential of 0 V versus RHE. Before linear sweep voltammetry and chronoamperometry measurements, the electrolyte was constantly bubbled with N_2_ to remove oxygen thereby eliminating erroneous signals arising from oxygen reduction.

## Conflict of Interest

The authors declare no conflict of interest.

## Supporting information

Supplementary
