# Supplementary material for: Earth‐Abundant Tin Sulfide‐Based Photocathodes for Solar Hydrogen Production
Source: Adv Sci (Weinh). 2017 Oct 16;5(1):1700362. doi: 10.1002/advs.201700362 (PMC5770675; doi:10.1002/advs.201700362)
Supplement: Supplementary file 1 — Supplementary [file ADVS-5-na-s001.pdf]

Copyright WILEY-VCH Verlag GmbH & Co. KGaA, 69469 Weinheim, Germany, 2017.

ADVANCED SCIENCE
Open Access

Supporting Information

for *Adv. Sci.,* DOI: 10.1002/advs.201700362

Earth-Abundant Tin Sulfide-Based Photocathodes for Solar Hydrogen Production

*Wei Cheng, Nirala Singh, Will Elliott, Joun Lee, Alan Rassoolkhani, Xuejun Jin, Eric W. McFarland, and Syed Mubeen**

Copyright WILEY-VCH Verlag GmbH & Co. KGaA, 69469 Weinheim, Germany, 2016.

Supporting Information

Title: Earth Abundant Tin Sulfide-Based Photocathodes for Solar Hydrogen Production

*Wei Cheng, Nirala Singh, Will Elliott, Joun Lee, Alan Rassoolkhani, Xuejun Jin, Eric W. McFarland, and Syed Mubeen**

Table S1. Comparison of H_2 production currents from SnS based photocathodes.

**Photocathode**	**Electrolyte**	**Light source (mW/cm^2)**	**IPCE**	**Photocurrent density (mA/cm^2)**
FTO/SnS/CdS/Pt[1]	0.5 M Na_2SO_4 (pH = 0.5)	100 (Sun simulator)	Not reported	0.7 at -0.4 V vs. Ag/AgCl
SnS/Pt[2]	0.1 M HCl + 10% Methanol	380 (UV light)	Not reported	~1.8 at -0.8 V vs. Ag/AgCl
SnS/NbO_2[3]	0.1 M Na_2SO_4	200 (660 nm LED)	Not reported	0.62 at -0.76 V vs. Ag/AgCl
SnS[3]	0.1 M Na_2SO_4	200 (660 nm LED)	10.8% at 400 nm	Not reported
SnS[4]	Na_2S + Na_2SO_3 (concentration is unkown)	Unknown (AM 1.5 M solar simulator with a UV cut-off filter)	Not reported	0.08 (without applied bias)
Present work	0.5 M H_2SO_4 (pH = 0)	80 (AM 1.5 solar simulator with 500 nm cut-off filter)	12.7% at 575 nm	2.4 at 0.22 V vs. Ag/AgCl

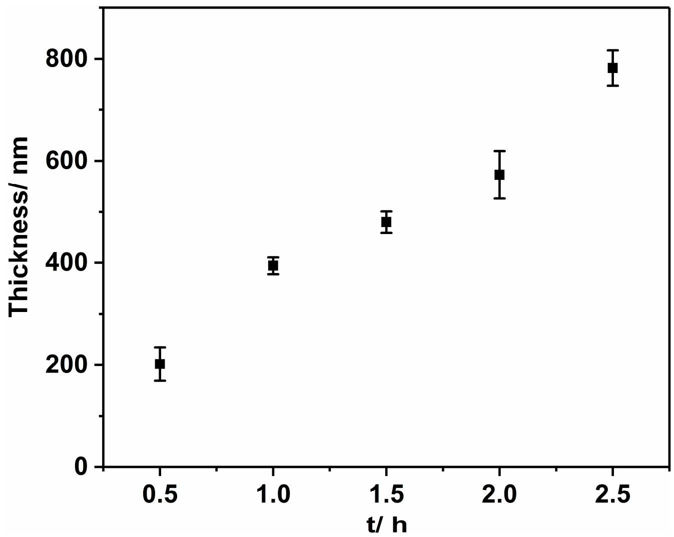

Figure S1. Dependence of SnS film thickness on the chemical bath deposition (CBD) time.

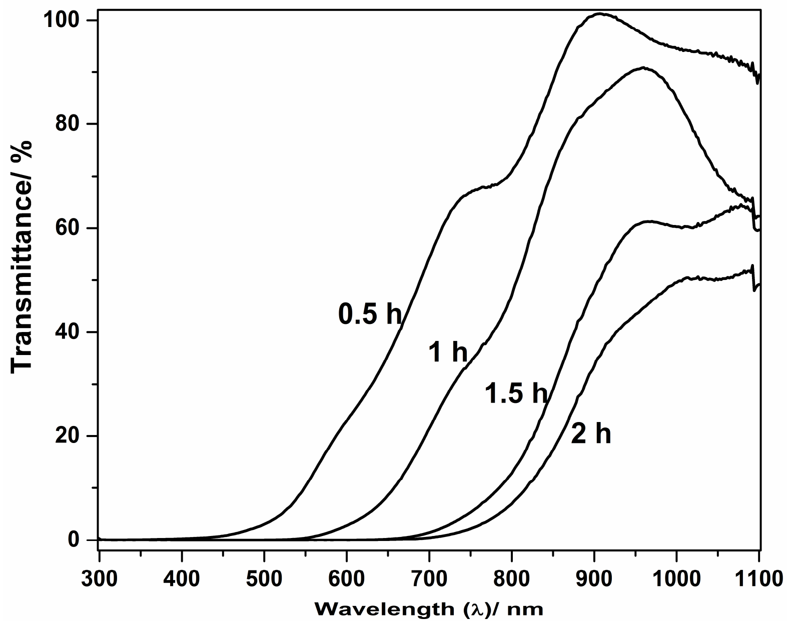

Figure S2. Optical absorption spectra of SnS films prepared via CBD for different deposition times (0.5, 1.0, 1.5, 2.0 h).

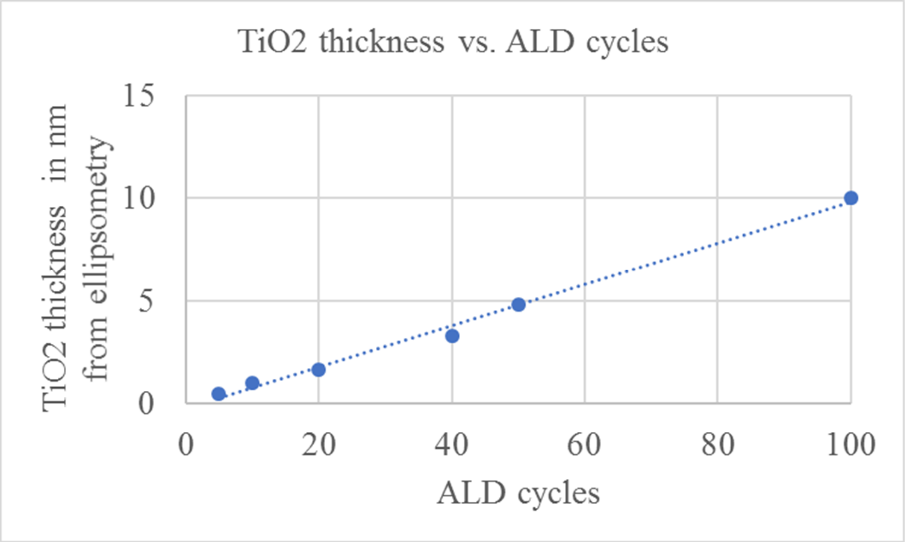

Figure S3. Thickness of TiO_2 films (obtained from ellipsometry) plotted as a function of ALD cycles. ALD of TiO_2 was carried out at 200℃ using tetrakis(dimethylamino)titanium (TDMAT) and water as precursor and reactant,

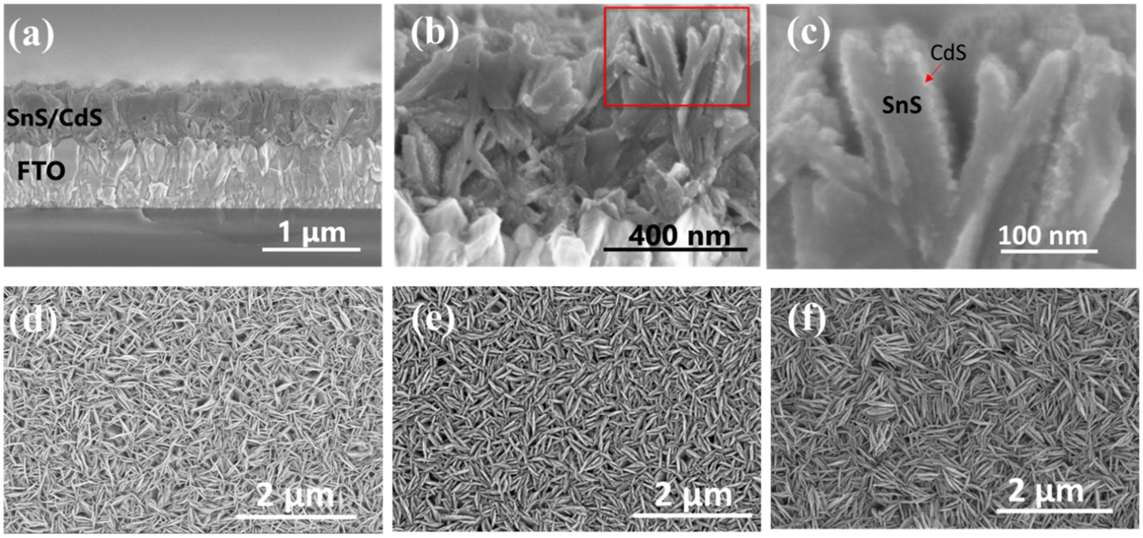

Figure S4. (a) Low (b) high magnification cross-sectional SEM images of SnS/CdS film. Figure S3c is the zoomed-in image of the part shown in the red box of Figure S3b. Low magnification top view SEM images of (d) SnS, (e) SnS/CdS and (f) SnS/CdS/TiO_2 films.

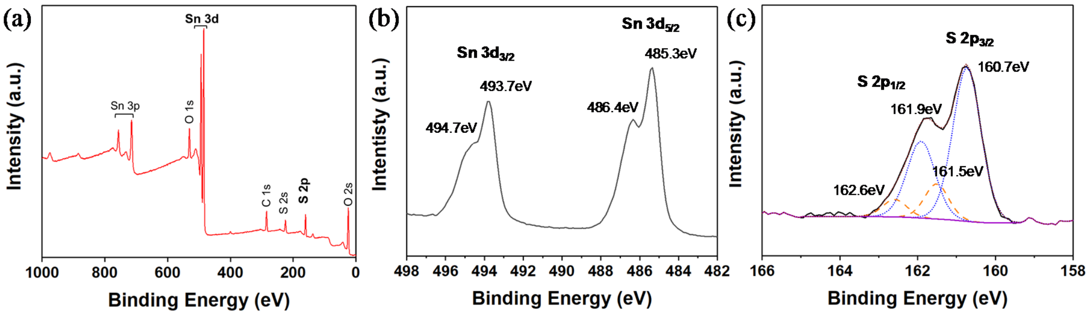

Figure S5. (a) Survey spectrum (b) – (c) core level spectra of Sn 3d and S 2p of SnS structures supported on a FTO glass substrate. The survey spectrum confirmed the presence of Sn and S showing intense signal for Sn 3d and S 2p. The Sn $3d_{5/2}$ core level spectrum showed two peaks at 485.3 and 486.4 eV, which correspond to the Sn-S bonds in the sample and the Sn-O bonds in the FTO substrate, respectively. The area ratio of the doublets of Sn $3d_{5/2}$ and $3d_{3/2}$ is 3:2 with separation of 8.4 and 8.3 eV for Sn-S and SnO, respectively. The S 2p core level spectrum showed two doublets, both refer to sulfide, with their $2p_{3/2}$ peaks located at 160.7 and 161.5eV.[5-6]

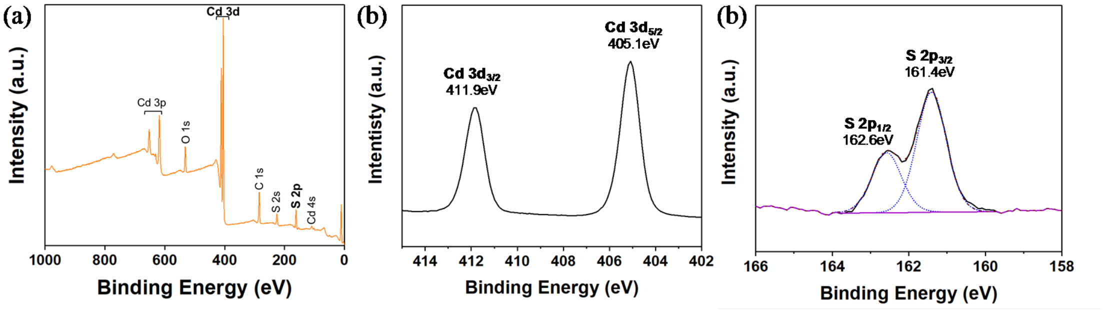

Figure S6. (a) Survey spectrum (b) – (c) core level spectra of Cd 3d and S 2p of SnS/CdS structures supported on a FTO glass substrate. The survey spectrum confirmed the presence of Cd and S showing intense signal for Cd 3d and S 2p. The Cd 3d core level spectrum showed sharp peaks at 405.1 and 411.9 eV, which correspond to $3d_{5/2}$ and $3d_{3/2}$ orbital levels, respectively. The doublet separation due to spin-orbit splitting is 6.8eV. The S 2p core level spectrum showed peaks at 161.4eV and 162.6eV for $2p_{3/2}$ and $2p_{1/2}$, respectively, with doublet separation of 1.2eV.[7]

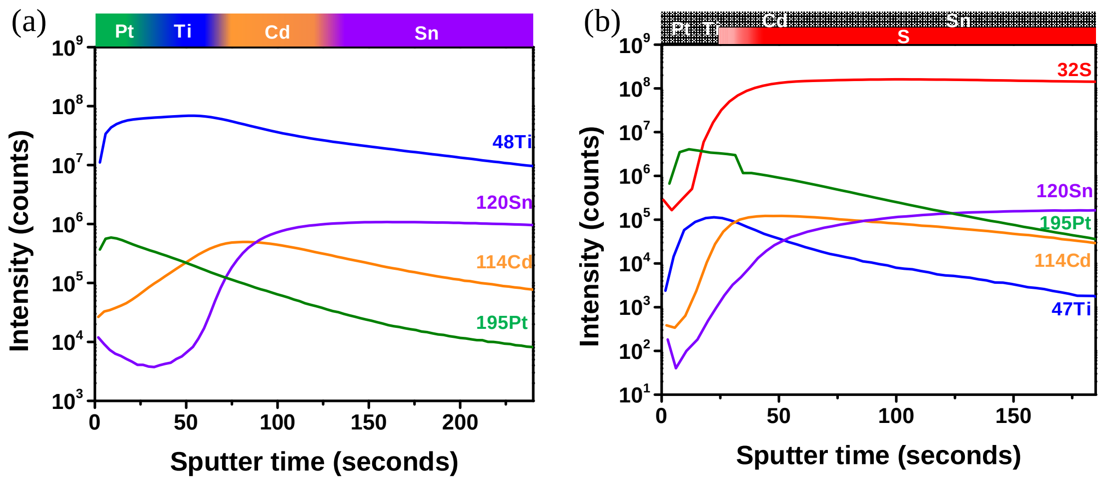

Figure S7. Depth profiles obtained using secondary ion mass spectrometry, confirming the proposed SnS/CdS/TiO_2/Pt structures with (a) positive ions for confirming metal strata and (b) negative ions to distinguish oxides and sulfides. The high surface roughness leads signal from upper layers dying out slowly.

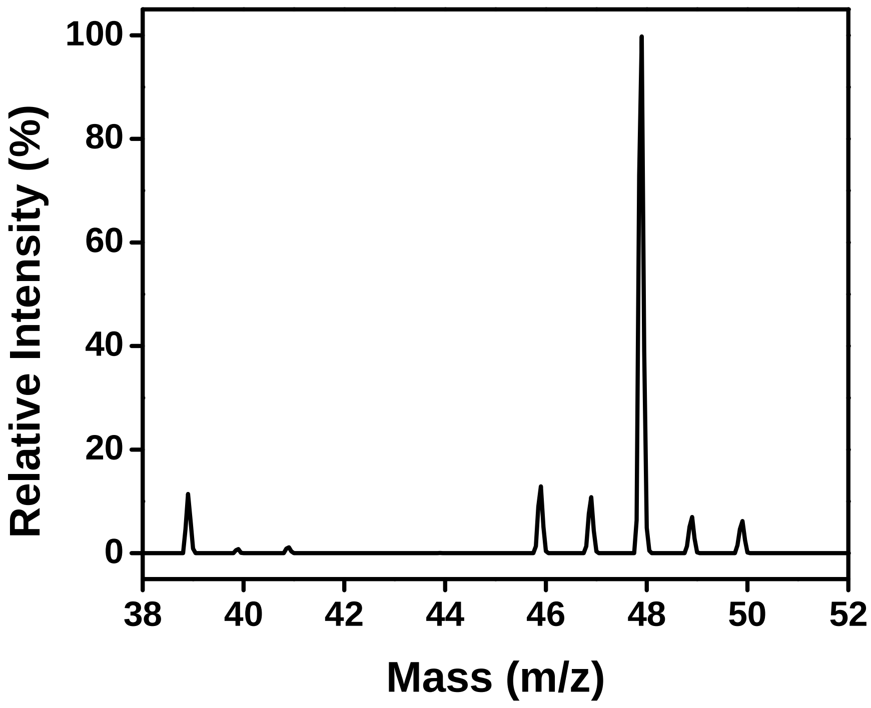

Figure S8. Positive-ion mass spectrum of film showing characteristic peaks of Ti from 46-50 m/z with relative intensities 46- 12.9%, 47 -10.8%, 48 - 100%, 49 – 7.0%, 50 – 6.21%, in good agreement with literature ratios[8], showing that signal at the 47 and 48 m/z peaks tracked in depth profiles correspond to Titanium.

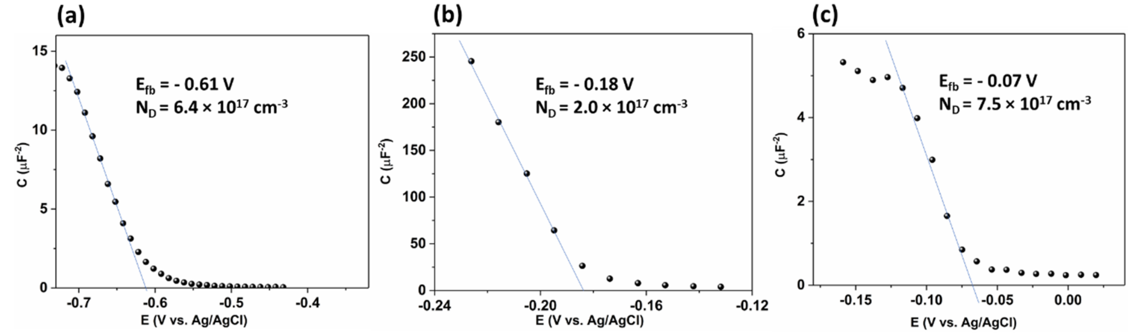

Figure S8. Mott-Schottky plots measured for (a) SnS (b) SnS/CdS and (c) SnS/CdS/TiO_2 films in 0.1 M Na2S + 0.1 M S (pH 9) at X frequency.

Figure S9. Mott-Schottky plots measured for (a) SnS (b) SnS/CdS and (c) SnS/CdS/TiO_2 films in 0.1 M Na2S + 0.1 M S (pH 9) at frequency of 8.3 kHz. The flat-band potential (Efb) and donor density (ND) values are provided as inset.

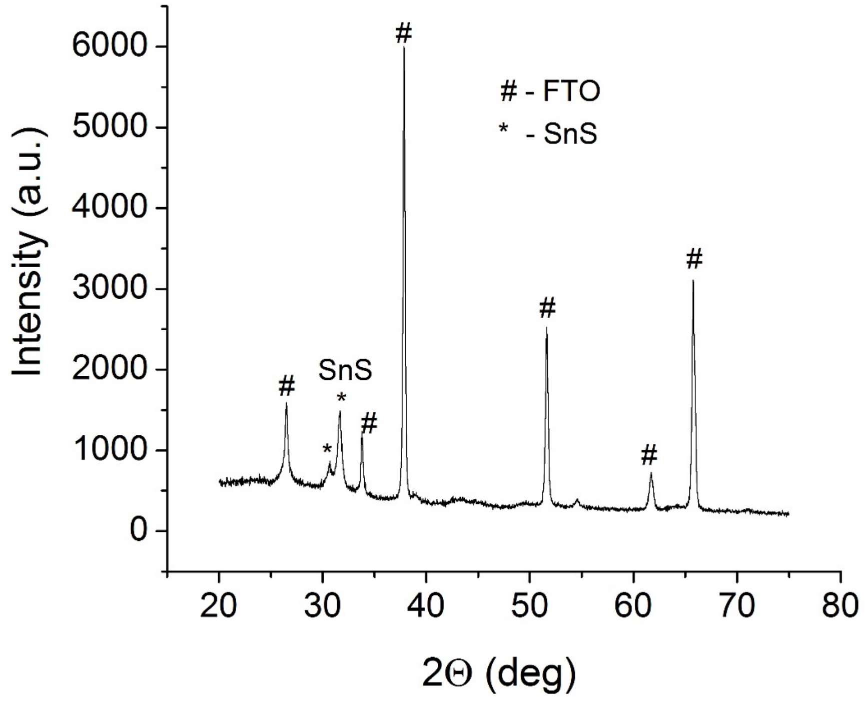

Figure S10. XRD of SnS/CdS/TiO_2/Pt after 3000s of PEC H_2 production run in 0.5 M H_2SO_4.

References:

[1] J. Jing, M. Cao, C. Wu, J. Huang, J. Lai, Y. Sun, L. Wang, Y. Shen, J. Alloys Compd. **2017**, 726, 720.
[2] M. Patel, X. Yu, Y. K. Kim, J. Kim, ChemNanoMat **2017**, 3, 591.

[3] J. J. M. Vequizo, M. Yokoyama, M. Ichimura, A. Yamakata, Appl. Phys. Express **2016**, 9.
[4] Y. Shiga, N. Umezawa, N. Srinivasan, S. Koyasu, E. Sakai, M. Miyauchi, Chem. Commun. **2016**, 52, 7470.
[5] J. Hou, C. Yang, Z. Wang, S. Jiao, H. Zhu, RSC Adv. **2012**, 2, 10330.
[6] C. Li, H. Zhang, C. Cheng, RSC Adv. **2016**, 6, 37407.
[7] P. Sinsermsuksakul, L. Sun, S. W. Lee, H. H. Park, S. B. Kim, C. Yang, R. G. Gordon, Adv. Energy Mater. **2014**, 4, 1400496.
[8] T.R. Hayes and J.F. Evans, J. Phys. Chem., **1984**, 88, 1963.
